# Surgical treatment of an aseptic fistulized acromioclavicular joint cyst: a case report and review of the literature

**DOI:** 10.4076/1757-1626-2-8388

**Published:** 2009-08-07

**Authors:** Luigi Murena, Fabio D’angelo, Daniele A Falvo, Ettore Vulcano

**Affiliations:** Department of Orthopaedic and Trauma Surgery, Insubria UniversityViale Borri 57, 21100 VareseItaly

## Abstract

An acromioclavicular joint cyst is an uncommonly reported condition, which seems to result from a massive rotator cuff tear and degenerative osteoarthritis of the acromioclavicular joint. We present the case of an 81-year-old man affected by an acromioclavicular joint cyst, associated to a massive rotator cuff tear, proximal migration of the humeral head and osteoarthritis of the gleno-humeral joint. The mass was 7 × 2.5 cm in size and the overlying skin presented a fistula that drained clear synovial-like fluid. Plain X-ray examination of the left shoulder showed proximal migration of the humeral head migration and osteoarthritis of the gleno-humeral joint, and further MRI evaluation confirmed the clinical diagnosis of a complete rotator cuff tear and observed a large subcutaneous cyst in communication with the degenerative acromioclavicular joint. The patient underwent surgical excision of the cyst and lateral resection of the clavicle to prevent disease recurrence. To the best of our knowledge, this is the first reported case of an acromioclavicular joint cyst complicated by an aseptic fistula resulting from multiple aspirations.

## Introduction

An acromioclavicular joint (ACJ) cyst is an uncommonly reported condition, which seems to result from a massive rotator cuff tear and degenerative osteoarthritis of the ACJ and gleno-humeral joint [1].

The most frequent presentation of this clinical entity is a painless, enlarging subcutaneous mass over the ACJ, which can often raise the suspicion of a tumor [2]. On arthrography the ACJ cyst typically presents with the “geyser sign”, but this technique has progressively been replaced by magnetic resonance imaging (MRI), which is currently the imaging technique of choice for the diagnosis of this pathology [3].

ACJ cysts can be managed either conservatively (observation, aspiration) or surgically, with or without rotator cuff repair, depending on several factors like age of the patient, symptoms and general health condition [2-6].

We report the case of man affected by an ACJ cyst associated to a massive rotator cuff tear that presented an aseptic cutaneous fistula secondary to multiple ultrasound-guided aspirations of the cyst.

## Case presentation

**Figure 1.**
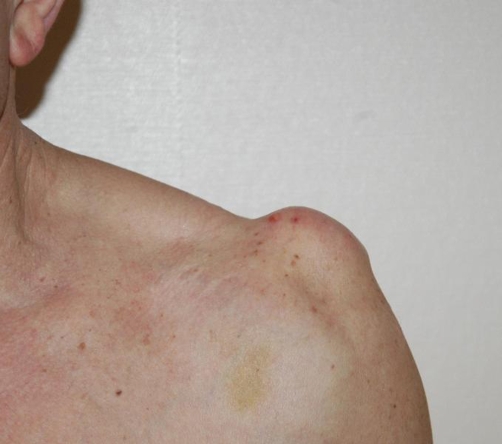
Preoperative image of the fistulized acromioclavicular joint cyst.

In November 2008 an Italian, Caucasian, 81-year-old male, a retired mason, presented to our outpatient clinic with a 1-year history of an enlarging subcutaneous mass over the left ACJ. The patient denied any previous shoulder trauma, but complained a long history of left shoulder discomfort during daily life activities. The patient had been submitted to four ultrasound-guided aspirations of the cyst and steroid injections by an interventional radiologist. After the final aspiration the patient complained recurrence of the cyst and formation of a fistula draining a slightly viscous serous fluid at the puncture site. On physical examination the mass was 7 × 2.5 cm in size, symmetric, firm, adherent to the superior aspect of the ACJ and non-tender on palpation (Figure 1). The overlying skin presented a fistula that drained clear synovial-like fluid. There was no associated lymphadenopathy. The active range of motion of the left shoulder was 160° in forward elevation, intrarotation was at the L2-L3 level, extrarotation and abduction of the arm were weaker with respect to the contralateral normal side, despite a normal range of motion.

**Figure 2.**
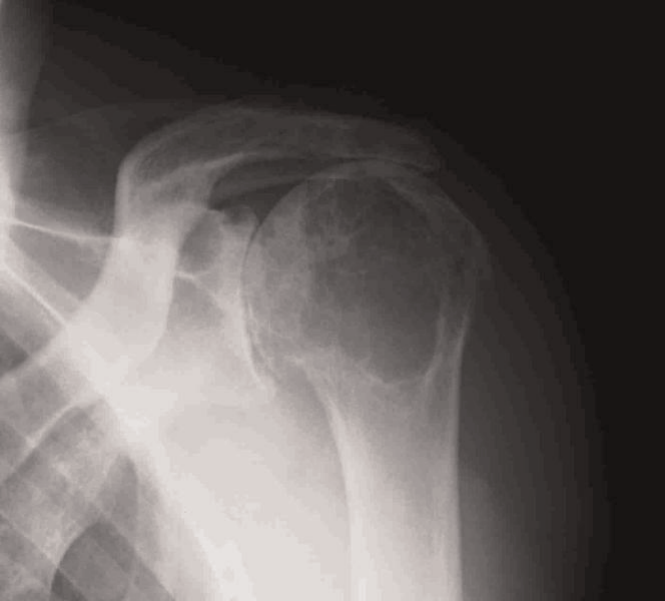
Preoperative shoulder X-ray.

**Figure 3.**
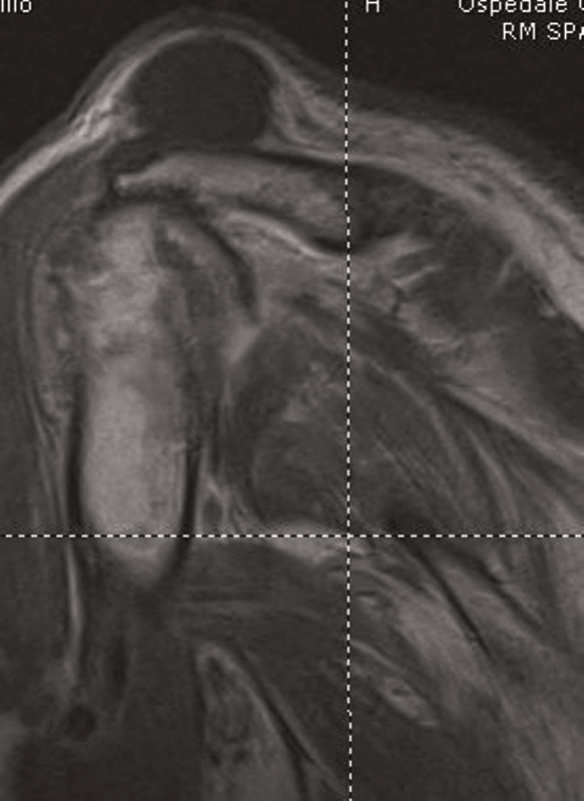
Preoperative shoulder MRI.

Plain X-ray examination of the left shoulder showed ACJ degeneration, with acromioclavicular (AC) space narrowing and osteophyte formation, proximal migration of the humeral head and osteoarthritis of the gleno-humeral joint (Figure 2). Further MRI evaluation confirmed the clinical diagnosis of a complete rotator cuff tear and observed a large, well-defined subcutaneous cyst in communication with the degenerative ACJ (Figure 3). Culture of the synovial-like fluid was negative and white blood cell count, erythrocyte sedimentation rate and reactive C-protein were within normal limits.

**Figure 4.**
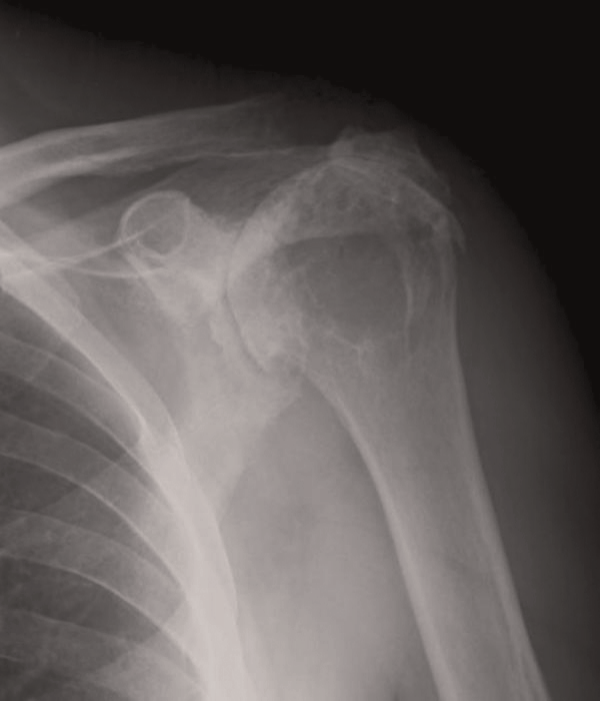
Postoperative shoulder X-ray.

In view of the persistence of the cyst and the draining fistula the we decided to submit the patient to surgery, which confirmed clinical and MRI findings. The cyst and the fistula were completely excised and, given the impossibility to repair the massively torn rotator cuff, to avoid recurrence of the cyst, resection of 1 cm of the lateral clavicle was also performed (Figure 4). Histological examination was suggestive of a synovial cyst.

Within three weeks from surgery function and range of motion of the operated shoulder returned to pre-operative levels. To date, the patient is asymptomatic and with no cyst recurrence.

## Discussion

ACJ cysts are rare clinical entities that seem to be secondary to traumas, connective tissue degeneration, and synovial extension from an adjacent joint [7-9]. Although some authors have observed no association between ACJ cysts and rotator cuff tear [10], most cysts seem to result from massive rotator cuff lesions [2] with proximal migration of the humeral head, and ACJ and gleno-humeral osteoarthritis. Craig *et al.* proposed that the ACJ cyst is formed by a leakage of synovial fluid through the torn rotator cuff and into a degenerated ACJ [3]. The fluid enters and distends the superior capsule of the ACJ, and elevates the skin [10]. Nardini *et al*. proposed a theory according to which mechanical irritation of the ACJ from direct contact of the humeral head in presence of a torn rotator cuff may lead to cyst formation [11].

The literature reports very few cases of ACJ cysts, most of which have been treated surgically, especially if associated to shoulder symptoms [2,4,5,7,10,12] Kessel *et al*. and Takagishi *et al*. demonstrated success of conservative treatment in half of patients with rotator cuff tear [13,14] Cvitanic *et al*. performed aspiration and corticosteroid injection of a cyst without success in terms of avoiding recurrence of the lesion [15]. Similarly, Postacchini *et al*. reported three ACJ cyst cases, one of which was treated with simple excision of the lesion that resulted in disease recurrence one year post-operatively [5]. Groh *et al*. managed four ACJ synovial cysts associated to gleno-humeral osteoarthritis and irreparable rotator cuff tear performing shoulder hemiarthroplasty, which proved to be an effective treatment [7]. Utrilla *et al*. performed excision of the cyst, acromioplasty and closure of the rotator cuff defect with a duramater allograft in a case of ACJ cyst associated to irreparable cuff tear with unsatisfactory functional results [16]. Le Huec *et al*. treated three patients with ACJ cysts and massive rotator cuff tears with excision of the cyst, removal of the distal clavicle end and synovectomy of the upper part of the pathological humeral-acromial joint [17]. At two-year follow-up no cyst recurrence was observed and the pain improved. Marino *et al*. besides excision of the cyst and lateral resection of the clavicle also repaired the torn cuff [4]. Arthroscopic treatment of ACJ cyst has also been recently described^2^. Unfortunately, there is no study reporting medium/long-term results of patients affected by ACJ cysts who underwent surgical excision along with rotator cuff repair, acromioplasty, lateral resection of the clavicle or hemiarthroplasty.

Treatment of ACJ cysts can be either conservative or surgical, according to symptoms, patient age, and shoulder function. In the presence of massive tear, few or no symptoms and good shoulder function in elderly patient conservative treatment is recommended. Conversely, patients with chronic shoulder pain and compromised shoulder function should be addressed to surgical treatment, to excise the cyst and possibly repair the torn cuff [7]. Groh *et al.* and Postacchini *et al.* clearly stated that without addressing the underlying pathological process, treatment of ACJ cysts proves to be unpredictable [5,7]. Nonetheless in the presence of an irreparable cuff tear resection of the lateral third of the clavicle reduces the risk of recurrences by removing the pinch-valve effect and is therefore recommended [2,5]. This was recently confirmed by Nowak *et al*. who reported the successful follow-up at 1 year in a patient with a massive ACJ cyst [1,2].

The present case reports an ACJ cyst in an elderly patient affected by rotator cuff arthropathy with negligible symptoms and a good function of the shoulder, that could be eligible for conservative treatment. However, the presence of a non-healing fistula draining synovial-like fluid, which significantly influenced quality of life of the patient, imposed an open surgical treatment. Hemiarthroplasty was not performed in view of the good function of the shoulder in absence of significant pain. To prevent cyst recurrence, we opted for lateral resection of the distal clavicle, in consideration of the irreparable rotator cuff tear.

To the best of our knowledge, this is the first reported case of an ACJ cyst complicated by an aseptic fistula resulting from multiple aspirations. Although aspirations of the cyst have been reported in the literature [14], in view of the high recurrence rate associated to this procedure and the increased risk of determining the formation of a fistula we discourage multiple ACJ cyst aspirations.

Also it is clear that the tendency is to surgically excise symptomatic ACJ cysts, along with the resection of the lateral clavicle. Although this procedure seems to be beneficial in terms of preventing disease recurrence, long-term follow-ups and report of more cases may help confirm the effectiveness of this surgical technique.

## Abbreviations

AC: acromioclavicular
ACJ: acromioclavicular joint
